# Supplementary material for: Comparison of kNN and k-means optimization methods of reference set selection for improved CNV callers performance
Source: BMC Bioinformatics. 2019 May 28;20:266. doi: 10.1186/s12859-019-2889-z (PMC6537193; doi:10.1186/s12859-019-2889-z)
Supplement: Supplementary file 1 — The file contains extended discussion and supporting data related to: (i) comparison to the reference set selection method proposed in CLAMMS and (ii) impact of chromosomal variability on the evaluation results. (PDF 771 kb) [file 12859_2019_2889_MOESM1_ESM.pdf]

Comparison of kNN and k-means optimization methods of reference set selection for improved CNV callers performance

Wiktor Kuśmirek, Agnieszka Szmurło,
Marek Wiewiórka, Robert Nowak, Tomasz Gambin

Comparison to the reference set selection method proposed in CLAMMS

The authors of CLAMMS [1] proposed a kNN based approach for reference sample set selection. In our work we have used similar algorithm and evaluated it's impact on the performance of CNV callers.

Although both methods rely on kNN algorithm, they use different distance metrics which affects final results of evaluation pipeline. To calculate the distances between each pair of samples CLAMMS recommends using a set of seven Picard (`http://broadinstitute.github.io/picard`) metrics:

- MEAN_INSERT_SIZE
- GC_DROPOUT
- AT_DROPOUT
- ON_BAIT_VS_SELECTED
- PCT_PF_UQ_READS

- PCT_TARGET_BASES_10X
- PCT_TARGET_BASES_50X

On the contrary, our kNN method utilizes the entire coverage profile i.e. vector of all coverage values, in building sample's neighbourhood. Next, in both methods the reference set for a given sample is constructed using its k nearest neighbours.

To compare the results of CLAMMS kNN algorithm based on Picard metrics, and our kNN algorithm based on coverage data we have performed the following analysis.

Firstly, we calculated Picard metrics for all 861 samples from the benchmark data set. Of note, this additional step required over 94 hours of computations. Then we obtained the correlation among the samples of the benchmark data set using multidimensional scaling (MDS) metric. The comparison of the results of MDS performed for Picard metrics and for full coverage profile (Supp. Fig. S1), revealed that our approach recovers the clustering structure of samples in 1000 genomes cohort better than the method implemented in CLAMMS. Evaluation of internal clustering metrics (Supp. Fig. S2) further confirmed this observation. Moreover these internal clustering metrics are inconsistent among each other, which make it difficult to select the optimal number of groups for k-means.

Finally, we compared the CODEX CNV detection performance while using three reference set selection methods, including kNN approach proposed by CLAMMS, kNN based on entire coverage profile and random selection of reference samples. Following CLAMMS' recommendations [1], we used a fixed number of 100 samples in each reference panel. The results revealed that among those three reference sample-set selection strategies, the kNN based on the full coverage profile leads to the best overall CNV detection performance (see Supp. Fig. S3).

Impact of chromosomal variability on the evaluation results

Due to the high computational complexity of some of the methods included in our benchmark and the large number of iterations of each test (e.g. for different k values in kNN) in the original evaluation we limited our analysis to the data from chromosome 1.

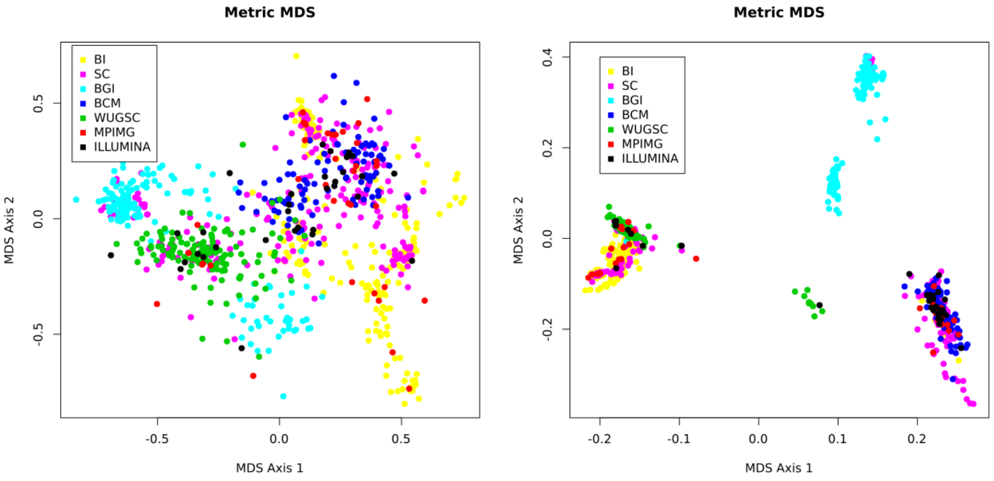

Figure S1: **Correlation between samples of benchmark dataset.** The graphs on the left and right present the diagrams for Picard metrics and full coverage profile, respectively.

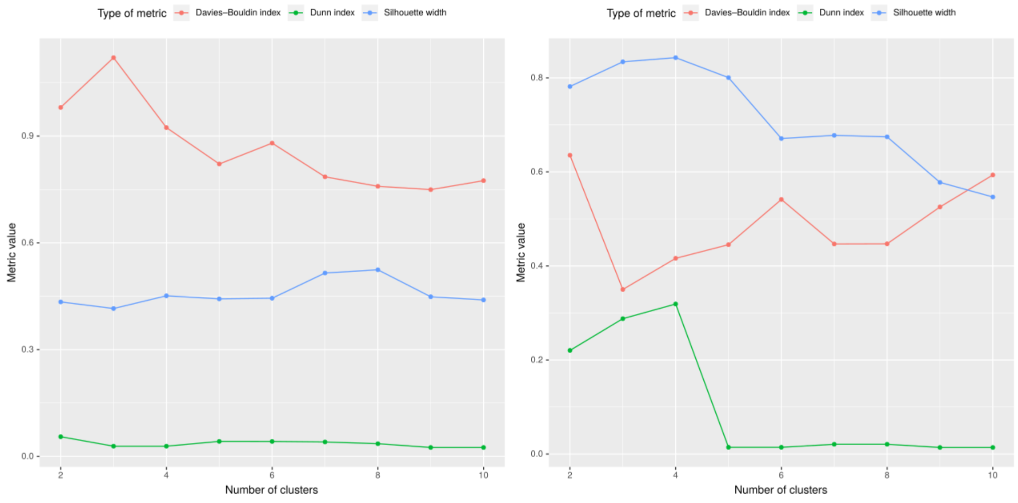

Figure S2: **Dunn index, Silhouette width and DaviesBouldin index for assessing the number of groups in k-means algorithm.** The graphs on the left and right present the diagrams for Picard metrics and full coverage profile, respectively.

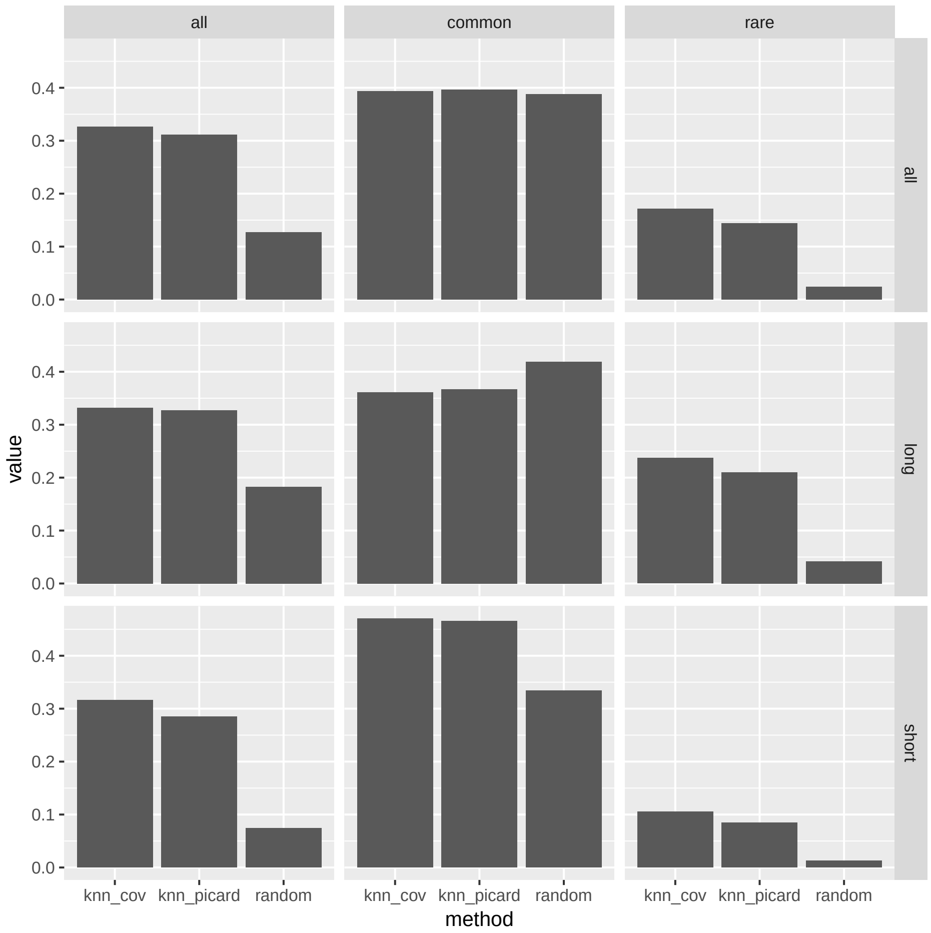

Figure S3: **F1 score of CNV calling using our kNN coverage-based approach (knn_cov), CLAMMS kNN Picard-based approach (knn_picard) and random selection (random).** Among those three reference sample set selection strategies, the overall CNV detection performance is the highest when using kNN based on full-coverage profile. The only exception when kNN Picard-based approach perform better than other solutions is detection rate of long, common CNVs which are however less important from the clinical perspective.

To assess the potential impact of chromosomal variability on our methods we repeated all analyses for another chromosome (chr11). First, we confirmed that the clustering structure recovered by MDS analysis for chr11 is very

similar to the one obtained for chr1 (Supp. Fig. S4). Analogously, we shown that the clustering internal metrics calculated for both chromosomes are virtually identical (Supp. Fig. S5).

Finally, the comparative analysis of different selection methods, various reference sample sizes, and three CNV calling methods was performed on data from chromosome 11 (Supp. Fig. S6). Although some results differ between chromosome 1 and 11, the general conclusions remain unchanged: (i) kNN and k-means clustering based reference selection methods improves the performance of CNV detection in comparison to "all" and "random" selections strategies; (ii) the results from "kNN" and "k-means" methods are highly similar, whereas the latter one is much less computationally extensive; (iii) the number of groups for k-means algorithm leading to the best CNV calling performance can be selected based on the analysis of internal clustering metrics.

The largest differences between chromosome 1 and 11 are observed in the precision and sensitivity of calling long CNVs (blue lines). The increased variability of CNV calling performance (e.g. peaks of blue line in Supp. Fig. S6C for CNVkit) can be explained by under-representation of large CNVs in chromosome 11.

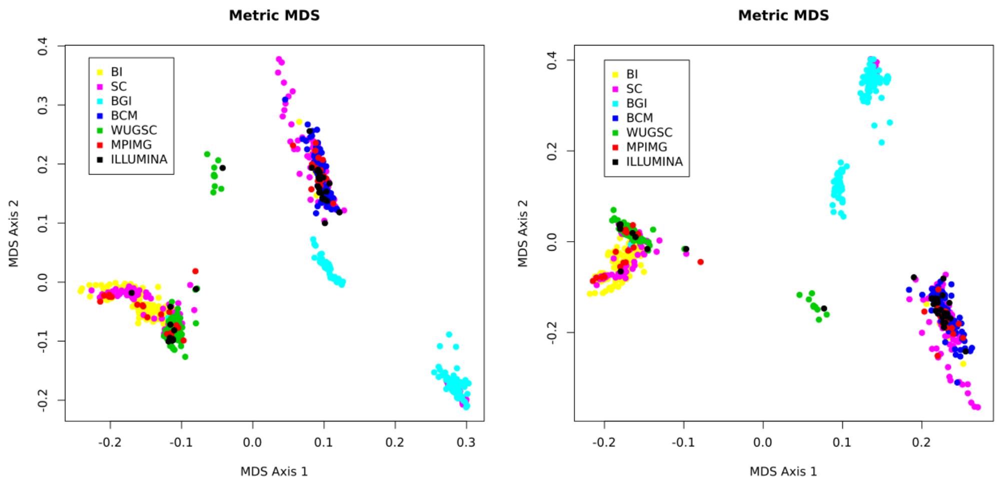

Figure S4: **Correlation between samples of benchmark dataset.** The graphs on the left and right present the diagrams for chromosomes 11 and 1, respectively.

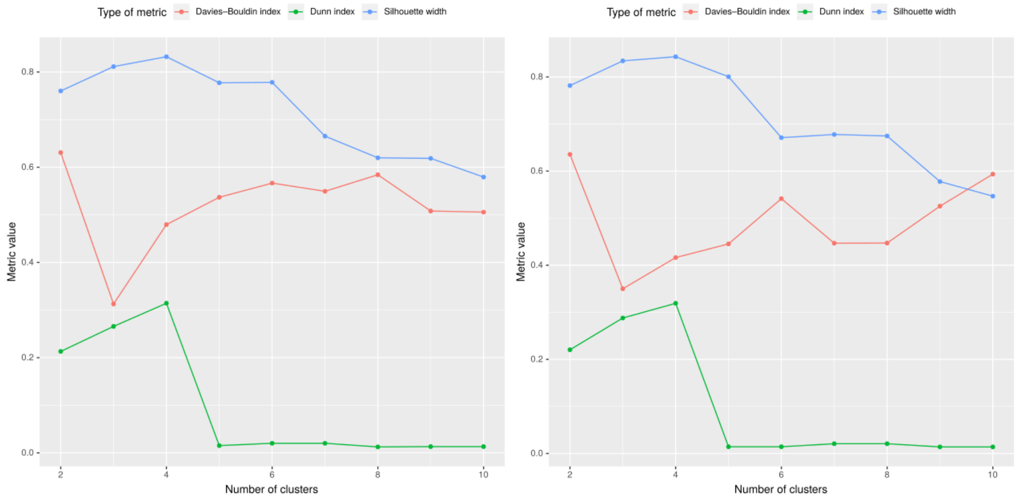

Figure S5: **Dunn index, Silhouette width and DaviesBouldin index for assessing the number of groups in k-means algorithm.** The graphs on the left and right present the diagrams for chromosomes 11 and 1, respectively. Importantly, for both chromosomes, two out of three metrics indicate the same optimal number of cluster (N=4).

References

[1] S Packer, J., K Maxwell, E., O'Dushlaine, C., E Lopez, A., E Dewey, F., Chernomorsky, R., Baras, A., D Overton, J., Habegger, L., G Reid, J.: Clamms: A scalable algorithm for calling common and rare copy number variants from exome sequencing data **32** (2015)

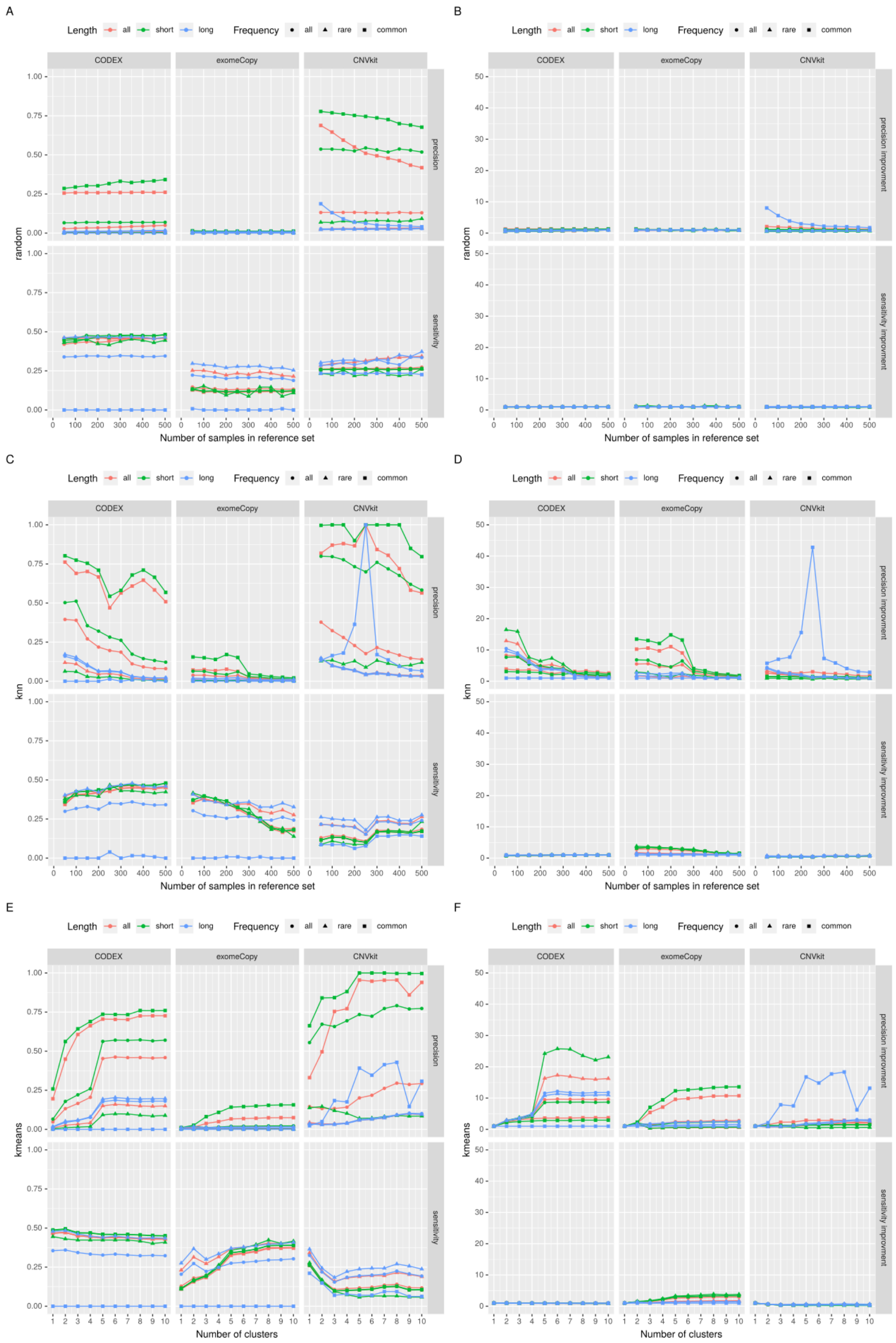

A
B
C
D
E
F
Length
all
short
long
Frequency
all
rare
common
CODEX
exomeCopy
CNVkit
precision
sensitivity
precision improvment
sensitivity improvment
random
knn
kmeans
Number of samples in reference set
Number of clusters

Figure S6: **Results of four selection method used in CODEX, CNVkit and exomeCopy for chr11.** Panels A, C, E present absolute changes in the precision and sensitivity of the investigated CNV callers for different methods of the reference set selection; relative performance in relation to baseline is presented in panels B, D, and F. The results for the "all" method (baseline) are presented in the "kmeans" diagram, where k is equal to 1 (single group). Major trends of CNV calling performance are consistent with the results obtained for chromosome 1. Although some differences between chromosomes (e.g. in the performance of calling long CNVs) arise due to under-representation of the specific class of variants in chromosome 11, they do not change the general conclusions.
